# Changes in food consumption and nutrition intake of rural residents in central China

**DOI:** 10.1016/j.heliyon.2024.e36523

**Published:** 2024-08-20

**Authors:** Ping Wen, Na Zhu, Mengmeng Jia

**Affiliations:** a School of Food Science, Henan Institute of Science and Technology, Xinxiang, 453003, China; b College of Tourism, Henan Normal University, Xinxiang, 453007, China

**Keywords:** Food consumption, Nutrition intake, Characteristic, Rural area

## Abstract

Food security and diet diversity is essential to sustainable food system all over the world. As the income of rural residents achieves great increase, the structure of food consumption and food diversification face significant change. Rural residents' food consumption and nutrients intake worth more attention. Chinese government has been striving to achieve sustainable development in rural areas. We conducted this study to explore area-level practices aiming at achieving food and nutrition security in rural China. In order to search for the change principle and main influencing factors of residents' food consumption, three rural areas in Henan Province were selected. According to the data obtained from the Henan Province Bureau of Statistics, changes in food consumption from 2012 to 2021 in three rural areas were analyzed in this study. This study led to the following remarkable results: (1) The per capita consumption of poultry, meat, sugar, and eggs of the three rural residents has increased much more than that of other food items. (2) In the three rural areas, the proportions of grains, vegetables, liquor, and edible oils have decreased overall. The proportions of other categories, such as poultry, meat, and fruits, have increased. (3) The rural residents’ per capita nutrition intake has increased remarkably. The results provide some empirical foundation for local government who need suggest that rural residents should control their intake of high-energy food, such as poultry, meat, and sugar. This study has significant policy implication for achieving sustainable goals in rural areas of China.

## 1 Introduction

In recent years, the concept of sustainability has been used not only in theoretical discussion but also in establishing environmental policies [1]. In the development of the food industry, it is very important to know of preferences of consumers, however, many factors can influence consumers' choices so the behavior of consumers becomes very complex [2]. With the development of living standards and the upgrading of residents’ consumption structure, rural residents are paying increasingly more attention to food quality and physical fitness. Meanwhile, the nutritional quality of Chinese diets has improved over time in some aspects, but worsened in others [3]. Diverse food is the basis of obtaining necessary micronutrients in required quantity [4]. Healthy diets can provide the necessary nutrients for people to prevent the disease and infection, the diversity of dietary is the basis of healthy diets and is vital to reduce the risks of diet-related diseases [[5], [6], [7]]. In one week, the intake of 20–30 types of food is helpful to healthy bodies of family member [8].

In recent years, the analysis of residents' nutritional health, dietary structure, eating behaviour, and related influencing factors has become a hot topic among scholars. With the development of economy, the meat consumption of residents achieves a growth to some extent [9,10]. And residents' food consumption actually is influenced by a series of factors, such as food price (economic factor), nutritional requirements (social factor), and crop planting area (ecological factor) [11].While, the nutrient intake's level hasn't got same improvement as food consumption quantity because of unsuitable food consumption structure [12]. These emergencies, such as pandemic, extreme weather, usually have vital impact on residents' food consumption structure and nutrients intake [13]. Moreover, the environmental impacts of food consumption patterns in different populations have been explored [3,[14], [15], [16]], and influencing their consumption behavior can minimize the impacts [17]. Health is an inevitable requirement for promoting the all-round development of humans. Food is closely related to human health, and the type, quantity, and quality of the food consumed by people every day have a significant impact on their health. Previous research has mainly focused on food consumption patterns [[18], [19], [20], [21]] and food consumption influence [[22], [23], [24], [25]]. Recently, many studies proved that the health–environment dilemma can be solved by changing the diet [26,27]. If reducing meat from their diets, households could cut much carbon emissions [28]. Modifying the consumption behavior of food, energy and water is vital to keep the sustainability of our earth [29]. With changes in industrialization, urbanization, an aging population, and the ecological environment, new challenges are being faced in maintaining and promoting health in China. China plans to significantly improve people's physical fitness by 2030, aiming to achieve an average life expectancy of 79 years. China is a country with a large population. In 2022, the rural population accounted for 36.11 % of the total population in China. With the continuous advancement of urbanization and the implementation of the rural revitalization strategy, the living standards of rural residents are constantly improving, and the dietary structure of rural residents has also undergone significant changes. Changes in the food consumption level and structure of rural residents have a huge impact on the security of arable land, the ecological environment, and sustainable development in China.

Despite many studies on food security have been conducted, the study focusing on the households in rural areas was ignored. This study aims to fill the gaps in the literature by examining the food consumption pattern and nutrition intake change. The objectives of this study were to analyze the food consumption and nutrition intake status and changes from 2012 to 2021, measure the change of energy and nutrition based on three rural areas. In China, the nutritional literacy level of rural residents is low, and the phenomenon of unbalanced diet is becoming increasingly prominent, resulting in overweight and obesity, diet-related chronic diseases, and micronutrient deficiencies. To evaluate the level of rural development, it is necessary to clarify the evolution of residents' food consumption and nutrition intake. Moreover, different rural areas need to be used as research objects, as more regional variations could be demonstrated. This study is to explore the sustainable practices for food consumption and nutrient security in different rural areas, aim to control the change in the residents' food consumption to reflect the change in the human–land relationship in rural areas, and help decision-making on rural sustainability, which plays a significant role in local sustainable development. This will also reveal the evolution characteristics of residents’ food consumption in terms of time and space, which could provide important insights for the formulation of local scientific and sustainable food strategies. This study can also provide implications for policymakers and health experts.

## 2 Methods

### 2.1 Study area

**Fig. 1:**
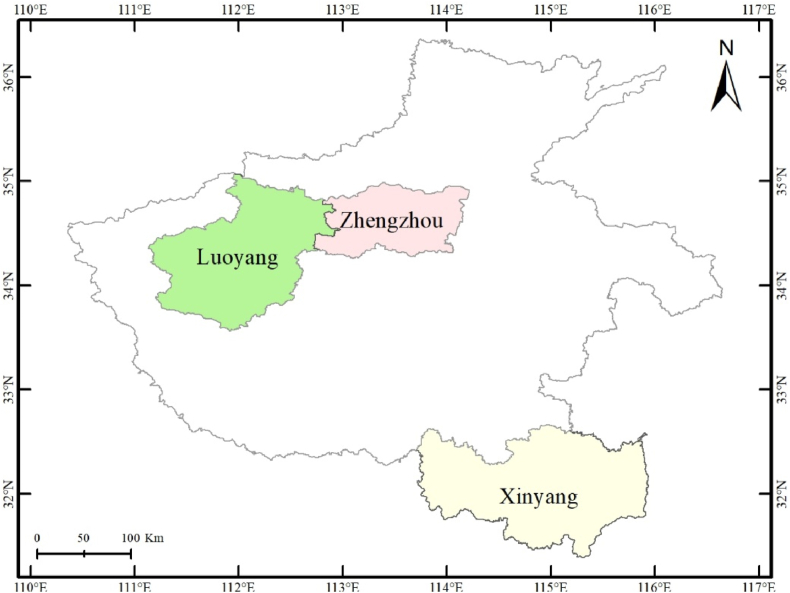
Administrative division map of Henan Province.

With a total size of 167000 km^2^, Henan is situated in central China at 31°23′ to 36°22′N and 110°21′-116°39′E, accounting for 1.73 % of China's total land area (Fig. 1). Plains account for 55.7 % of the total area in Henan Province. The warm temperate climate, with four distinct seasons and simultaneous heat and precipitation, covers the majority of the region. In the past 10 years, the average annual temperature in Henan has been 15.1°C–15.9 °C, the average annual precipitation has been 512.6–1129.1 mm, and the average annual sunshine hours have been from 1774.5 to 2024.1 h. In 2022, the registered population in Henan was 98.72 million people; among them, the urban population was 56.33 million people, and the rural population was 42.39 million people. The urbanization rate of household registration was 57.07 %. In 2022, the gross regional product of the province was 912.04 billion U.S. dollar, the industrial added value was 291.29 billion U.S. dollar, and the total retail sales of consumer goods was 362.88 billion U. S. dollar. In comparison to 2021, the per capita disposable income of the inhabitants increased by 5.3 %–4195.89 U S. dollar, urban residents' per capita disposable income increased by 3.7 %–5721.59 U S. dollar, rural residents' per capita disposable income increased by 6.6 %–2779.77 U S. dollar.

Henan is the core area of national grain production, the main province of pig and poultry production, and an advantageous production area for large cash crops. The combined value of the output from fisheries, forestry, agriculture, and animal husbandry exceeds 148.68 billion U.S. dollar, ranking second in China. The annual grain planting area in Henan Province is 10778.35 thousand hectares, including a wheat planting area of 5682.45 thousand hectares, a corn planting area of 3857.52 thousand hectares, an oil planting area of 1592.45 thousand hectares, and a vegetable planting area of 1782.5 thousand hectares. The wheat yield and seed production capacity both rank first, while the oil and vegetable yields rank first and second in China, respectively.

Henan is a major agricultural province and a microcosm of China, produces one tenth of the country, and shoulders the responsibility of ensuring China's food security. Henan Province ranks among the top in population in China, and rural population accounts for 43 % in 2022. Rural residents' food security plays the vital role in China's food security. Studying the food consumption and nutritional intake of rural residents in Henan Province has reference significance for understanding the overall food consumption situation of rural residents in China. Zhengzhou, Luoyang, and Xinyang are representative cities of Henan Province. Xinyang is located in the southernmost part of Henan Province, at the north-south boundary of China, and receives more precipitation than other parts of Henan Province, with rice as the main crop and the main diet. And the diet habit in Xinyang is the representative of southern Henan. As the ancient capital of the Nine Dynasties, Luoyang was once the political, economic, and cultural center of China. Luoyang has a high wheat yield, mainly consisting of pasta and soup dishes. Zhengzhou is the capital city and economic center of Henan, the diet habit is the representative of northern Henan.

Zhengzhou, the capital city of Henan Province, is located in the central–northern part of Henan Province (Fig. 1). The basic information of three cities has been showed clearly (Table 1). The total area of Zhengzhou is 7567 km^2^. In 2022, the permanent population of Zhengzhou was 12.83 million, its regional GDP was 192.31billion U.S. dollar, and the per capita disposable income of residents for the year was 6102.94 U S. dollar; among them, in comparison to 2021, the per capita disposable income of rural residents was 4198.12 U S. dollar, the annual per capita living expenses of rural residents increased by 2 %–3937.50 U S. dollar, the per capita living consumption expenditure of rural residents increased by 3.1 %–3045.45 U S. dollar.

**Table 1:** The basic information of study area.

District	Henan province	Zhengzhou city	Luoyang city	Xinyang city
Area	167000 km^2^	7567 km^2^	15230 km^2^	18916 km^2^
Permanent population (2022)	98.72 million	12.83 million	7.08 million	6.166 million
Gross regional product (U.S. dollar)	912.04 billion	192.31billion	84.38 billion	47.52 billion
Per capita disposable income (U.S. dollar)	4195.89	6102.94	4696.03	3772.77
Per capita disposable income of rural residents (U.S. dollar)	2779.77	4198.12	2721.64	2664.69
Per capita living consumption expenditure of residents (U.S. dollar)	2830.21	3937.50	3302.51	2648.93
Per capita living consumption expenditure of rural residents (U.S. dollar)	2205.95	3045.45	2158.75	2129.61

Luoyang is located in the western part of Henan Province, with a total area of 15230 km^2^ (Fig. 1). In 2022, the permanent population of Luoyang was 7.08 million, its regional GDP was 84.38 billion U.S. dollar, and the per capita disposable income of residents in the city for the whole year was 4696.03 U S. dollar; among them, the per capita disposable income of rural residents was 2721.64 U S. dollar, and the per capita consumption expenditure of residents was 3302.51 U S. dollar, an increase of 6.1 % compared to that in 2021. The per capita consumption expenditure of rural residents was 2158.75 U S. dollar, an increase of 4.8 %.

Xinyang is located in the southern part of Henan Province, with a total area of 18916 km^2^ (Fig. 1). In 2022, the permanent population of the city was 6.166 million, comprising 3.19 million urban residents and 2.97 million rural residents. In 2022, the regional GDP was 47.52 billion U.S. dollar. The per capita disposable income of residents for the year was 3772.77 U S. dollar; among them, the per capita disposable income of rural residents was 2664.69 U S. dollar, with an increase of 8.0 %. The per capita living consumption expenditure of residents was 2648.93 U S. dollar. The per capita living consumption expenditure of rural residents was 2129.61 U S. dollar, with an increase of 8.0 %.

### 2.2 Methods

In this study, the main data on the consumption of food, such as grains, edible oils, vegetables, meat (pork, beef, and mutton), poultry, aquatic products, eggs, milk, fruits, and sugar, were obtained from the official statistics website of Henan Province Bureau of Statistics (https://tjj.henan.gov.cn/). Furthermore, development statistics, such as per capita disposable income, population, and the planting area of grain, were obtained from the official website. To evaluate the changes in and nutrition intake of residents’ food consumption in different rural areas, conversion factors for different food items were applied (Table 2). The specific equations and explanations were as follows:

(1) $$C_{f}=\sum_{i=1}^{n}x_{i}\times r_{p}$$

where $C_{f}$ is the total quantity of per capita food consumption; $x_{i}$ is a specific food item, such as grains, edible oils, and vegetables; and $R_{p}$ is the rural population.

(2) $$N_{i}=\sum_{i=1}^{n}x_{i}\times r_{i}$$

where $x_{i}$ is a specific food item, such as grains, edible oils, and vegetables; $r_{i}$ is the conversion factor for different food items per 100g, such as energy, protein, fat, and carbohydrates; and $N_{i}$ is the summation of nutrient acquisition from each specific food.

**Table 2:** The Conversion factor for different food items per 100g

Number	Food Item	Energy (kcal)	Protein (g)	Fat (g)	Carbohydrates (g)
1	Grains	344	11	1.3	74.3
2	Edible oils	899	0	99.9	0
3	Vegetables	28	1.8	0.2	4.2
4	Meat	235	16.2	24	2.4
5	Poultry	152	17.3	8.9	1.3
6	Aquatic products	116	20.1	4.2	0.2
7	Egg	156	12.8	11.1	1.3
8	Milk	54	3	3.2	3.4
9	Fruits	41	0.2	0.2	12.3
10	Sugar	289	8.3	5.1	58.1
11	Liquor	297	0	0	0

## 3 Results analysis

### 3.1 Changes in per capita food consumption in the rural areas of Zhengzhou, Luoyang, and Xinyang

**Fig. 2:**
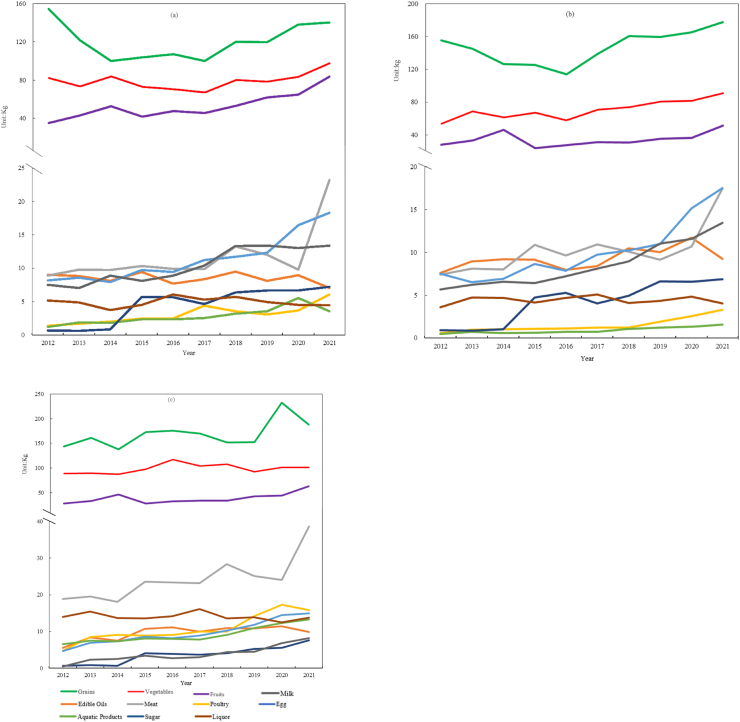
Changes in per capita annual food consumption in three rural areas: (a) Zhengzhou; (b) Luoyang; (c) Xinyang.

In this section, the change and its characteristics of per capita food consumption of rural residents in Zhengzhou, Luoyang and Xinyang are analyzed and compared from 2012 to 2021 (Fig. 2). It is found that the consumption of grains, vegetables, and fruits is higher than that of other food types in these three rural areas. The per capita consumption of grains by rural residents is slightly higher in Luoyang and Xinyang than that in Zhengzhou. The per capita consumption of vegetables by rural residents in Xinyang and Zhengzhou is higher than that in Luoyang. The per capita consumption of fruits by rural residents in Zhengzhou is significantly higher than that in Xinyang and Luoyang.

During the 10-year study period, the per capita consumption of meat (such as pork, beef, and mutton) by rural residents increased significantly. The growth rates in the rural areas of Zhengzhou, Luoyang, and Xinyang from 2012 to 2021 were 160 %, 137 %, and 104 %, respectively. However, in 2020, there was a significant decrease in the rural areas of Zhengzhou and Luoyang, which was mainly due to the impact of the COVID-19 pandemic. The per capita consumption of eggs and their products by rural residents also significantly increased. The growth rates of the three rural areas of Zhengzhou, Luoyang, and Xinyang from 2012 to 2021 were 123 %, 134 %, and 219 %, respectively. The per capita consumption of sugar by rural residents significantly increased, with the growth rates of Zhengzhou, Luoyang, and Xinyang being 929 %, 666 %, and 983 % from 2012 to 2021, respectively. The per capita consumption of milk by rural residents also significantly increased, especially in Xinyang, with a growth rate of 15 times, with Zhengzhou and Luoyang accounting for 79 % and 136 %, respectively. The per capita consumption of poultry by rural residents increased significantly, with the growth rates in Zhengzhou, Luoyang, and Xinyang being 332 %, 452 %, and 229 %, respectively.

**Fig. 3:**
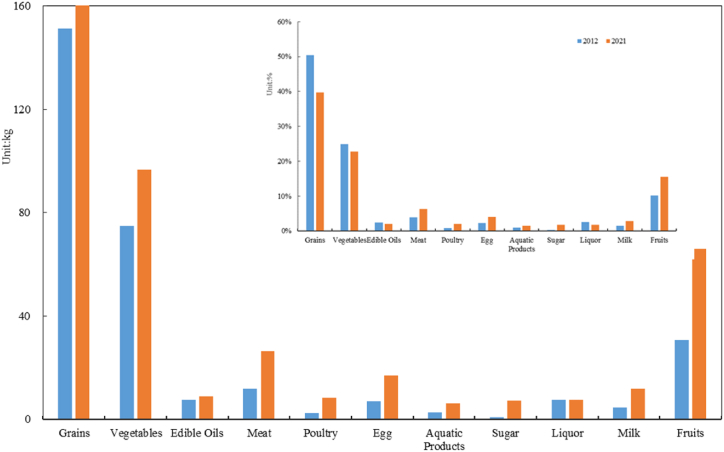
Food consumption structure and change (based on the average data of three rural areas in 2012 and 2021).

Next, we understand the quantity and structure of various food consumptions per capita per year during the study period. When comparing the food consumption across the entire study period, it could be seen that the proportion of grains, vegetables, alcohol, and edible oils decreased overall (Fig. 3). The proportion of grain consumption in 2012 was 50.3 %, and, in 2021, it was 39.8 %. The proportion of other categories, such as poultry, meat, and fruits, increased. For example, fruits accounted for 10.2 % of the year's food consumption in 2012 and 15.6 % in 2021. This means that, with the development of the economy and the transformation of rural residents' consumption concepts, rural residents' food consumption types became more diversified, and their nutrient intake channels became more diverse.

### 3.2 Changes in nutrient intake from food consumption

#### 3.2.1 Per capita nutrient intake level in the rural areas of Zhengzhou, Luoyang, and Xinyang

##### 3.2.1.1 (1) energy

**Fig. 4:**
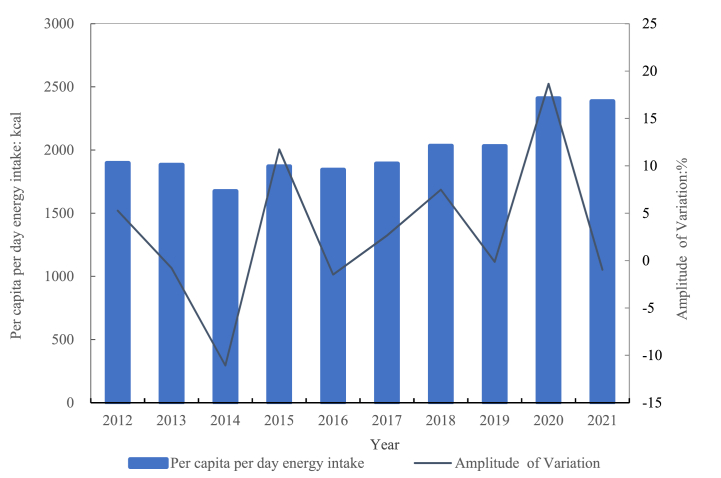
Changes in three rural areas per capita per day energy intake.

In this study, to better measure the food consumption and energy intake of rural residents in three different cities over the past 10 years, the average daily energy intake per person in three rural areas in 2012 and 2021 was selected for plotting. It could be seen that there was an overall rising trend (Fig. 4). In 2012, each person consumed about 1896.9 calories of energy every day. In 2013, the energy intake decreased by 0.78 % compared to the previous year. In 2014, there was a small decrease, but it wasn't very big, with a decrease of 11.74 %–1673.7 kcal per person per day. In 2018, the per capita energy intake exceeded 2000 kcal per day, reaching 2032.8 kcal per day. In 2020, the daily average per capita intake reached its highest level in a decade, at 2408.9 kcal.

**Fig. 5:**
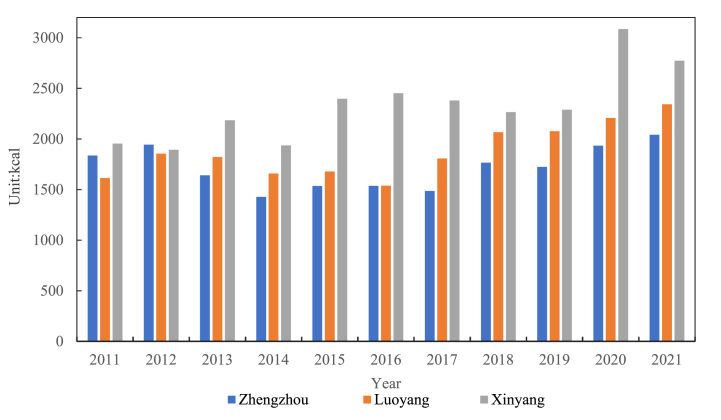
Changes in per capita per day energy intake in different rural areas.

There were a lot of different ways that people's energy intake changed in different places. Among the three selected rural areas, the overall energy intake of the Xinyang rural area was higher than that of the Zhengzhou and Luoyang rural areas (Fig. 5). The change trends of the Zhengzhou and Luoyang rural areas were the same, with a lower intake in 2013–2017 and a higher intake in the first and last examined years. The highest daily average energy intake per person in the Zhengzhou rural area was 2041.8 kcal in 2021, and the lowest was 1426.8 kcal in 2014. In 2012, the average energy intake in the Luoyang rural area was 1855.2 kcal/capita/day, the lowest value was 1539.2 kcal in 2016, and the highest value was 2342.5 kcal in 2021. The average daily energy intake per person in the Xinyang rural area fluctuated over the past decade, showing an increasing trend overall. In 2012, the average energy intake was 1892.5 kcal/capita/day, lower than the 1943.3 kilocalories in the Zhengzhou rural area in the same year. In 2020, the average energy intake exceeded 3000 kcal/capita/day, with a value of 3085.1 kcal.

##### 3.2.1.2 (2) protein, fat, and carbohydrates

**Fig. 6:**
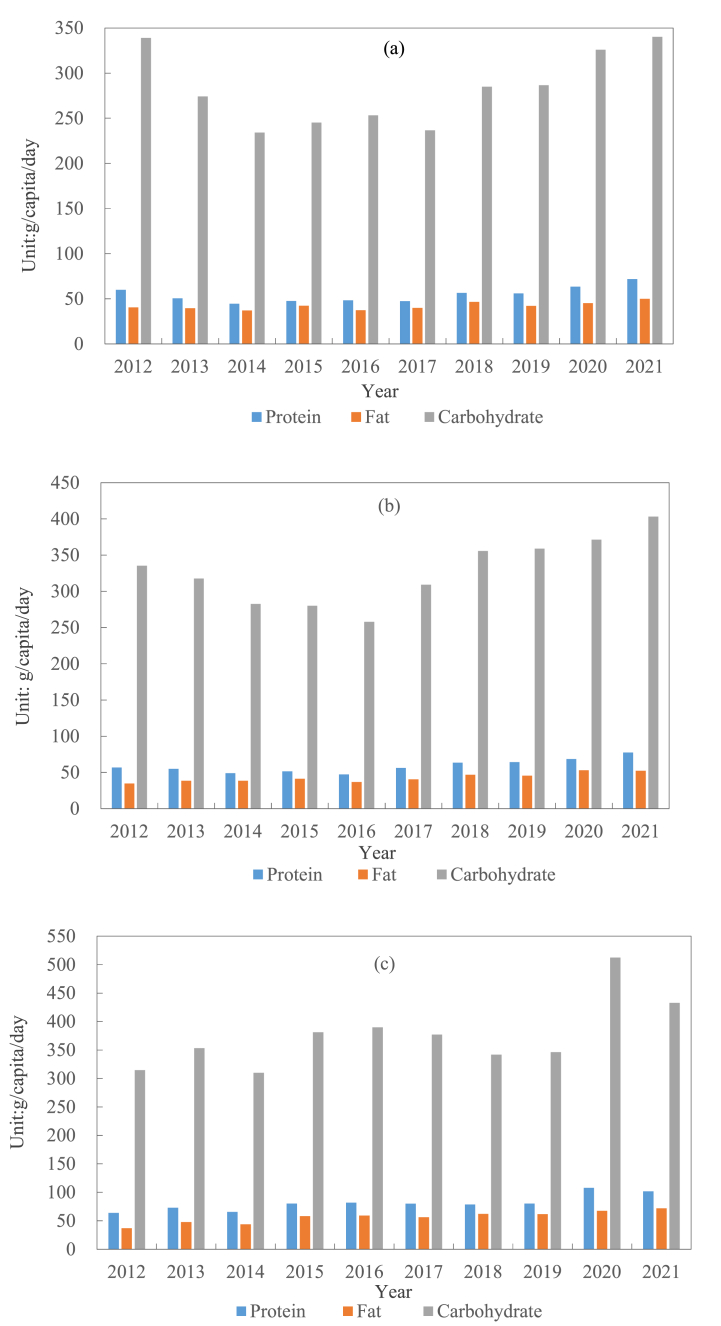
Per capita per day intake of protein, fat, and carbohydrates in different rural areas: (a) Zhengzhou rural area; (b) Luoyang rural area; (c) Xinyang rural area.

The changes in the carbohydrate, protein, and fat intake per capita per day in the rural areas of Zhengzhou, Luoyang, and Xinyang were different. In terms of carbohydrate intake, the changes in the rural areas of Zhengzhou and Luoyang were the same, showing a valley-shaped distribution, with a lower intake in the years in the middle of the study period and a higher intake in the first and last examined years (Fig. 6). The carbohydrate intake in the Zhengzhou rural area per capita per day in 2012 and 2021 was 339g and 340.3g, respectively, which were almost the same. From 2013 to 2019, the average annual average was below 300g per day, with a minimum value of 234g in 2014. The changing trend of the Luoyang rural area was the same as that of the Zhengzhou rural area. From 2012 to 2016, the data kept declining, from 335.2g in 2012 to 257.9g in 2016. From 2017 to 2021, it continued to grow, breaking through 400g and reaching 403.2g in 2021, an overall increase of 20.3 % compared to 2012. The overall average carbohydrate intake per person per day in the Xinyang rural area showed a fluctuating trend. It was 314.6g in 2012; exceeded 500g in 2020, reaching the peak within 10 years; and dropped to 432.8g in 2021, which might be related to the impact of COVID-19 in 2020, when people spent most of their time at home and consumed more staple foods.

The overall intake of fat and protein in the rural areas of Zhengzhou, Luoyang, and Xinyang was lower than that of carbohydrates, showing an upward trend. The average intake of protein per capita per day in the Zhengzhou rural area was 59.8g in 2012 and 71.9g in 2021. In the Luoyang rural area, the daily average intake of protein per capita was 56.8g in 2012 and 77.3g in 2021. In the Xinyang rural area, the daily average intake of protein per capita was 63.9g in 2012 and 101.7g in 2021, being the only rural area that exceeded 100g. In terms of fat intake, the daily average intake per person in the Zhengzhou rural area was 40.4g in 2012 and 50.1g in 2021, an increase of about 25 % compared to 2012. In the Luoyang rural area, the daily average intake per capita was 34.8g in 2012, which continued to increase from 2012 to 2015, decreased to 36.8g in 2016 compared with the previous year, and increased to 52.5g in 2021. In the Xinyang rural area, the daily average intake per capita was 37.0g in 2012, which decreased to 43.7g in 2014 compared with the previous year. From 2015 to 2021, the daily average intake per capita increased and reached 71.9g in 2021.

#### 3.2.2 Per capita nutrient intake in different rural areas

##### 3.2.2.1 (1) energy

**Fig. 7:**
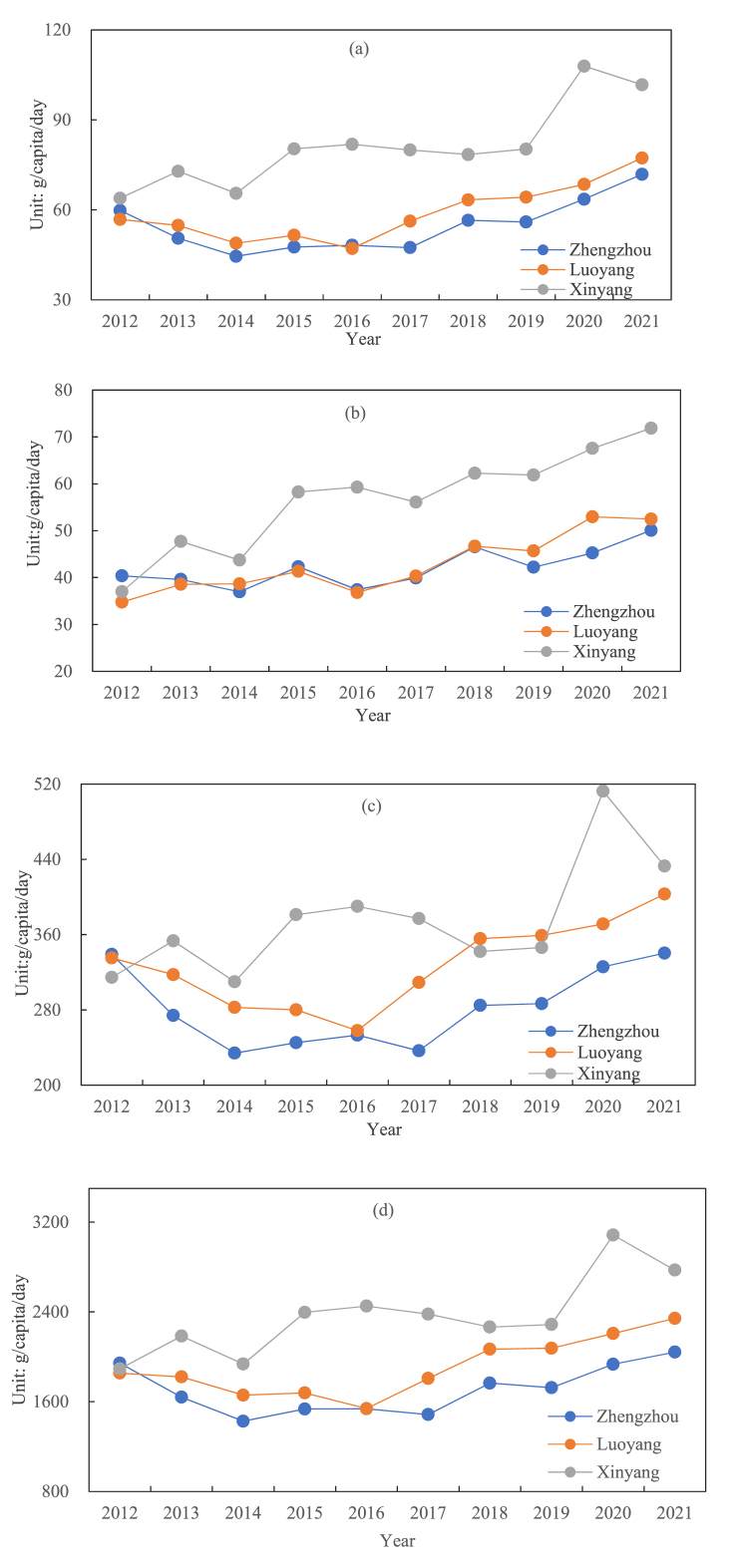
Changes in per capita daily nutrient intake in different rural areas: (a) protein; (b) fat; (c) carbohydrates; (d) energy.

After comparing and analyzing the per capita energy intake in the three rural areas, although the per capita energy intake in the three places increased in the later years compared to the first years of the study period, there were still big changes that happened over time. (Fig. 7). During the study period from 2012 to 2021, the daily energy intake per capita of the Xinyang rural area was much higher than that of the other two rural areas, especially in 2020, where the daily energy intake per capita of the Xinyang rural area was more than 1100 calories higher than that of the Zhengzhou rural area and more than 800 calories higher than that of the Luoyang rural area. According to the Chinese Dietary Guidelines in 2022, the recommended per capita energy intake is 1600–2400 kcal.

##### 3.2.2.2 (2) protein, fat, and carbohydrates

Xinyang rural residents eat more protein, carbohydrates, and fat each day compared with that in Zhengzhou and Luoyang rural area. In terms of the daily average protein intake per capita, the overall trends of these two rural areas of Zhengzhou and Luoyang in the initial stage of the study (2012–2016) were downward, and they have been rising steadily since 2016 and 2017, respectively. The average daily protein intake of the Zhengzhou rural area and the Luoyang rural area in the past 10 years was 54.6g and 58.9g, respectively, and that of the Xinyang rural area was 81.3g. The data on fat intake over the past decade showed that the changes in the Zhengzhou rural area and the Luoyang rural area fluctuated upwards and downwards, with an overall upward trend. The daily average intake of fat per capita in the two rural areas increased by 25 % and 50 % in 2021, respectively, compared with 2012, and that in the Xinyang rural area increased by 94 % in 2021 compared with 2012. Finally, carbohydrates, as one of the most important intake substances, its overall changes in the three rural areas over the past decade showed significant fluctuations. The lowest intake in the rural areas of Zhengzhou, Luoyang, and Xinyang occurred in 2014 (234g), 2016 (257.9g), and 2014 (309.9g), respectively. The carbohydrate intake of the Xinyang rural area reached the highest in 2020, much higher than that in the other years, reaching 512.4g, an increase of 48 % compared with 2019, and it decreased to 432.8g in 2021.

## 4 Discussion

With the development of rural living standards, rural residents focus much more on the per capita intake of vegetables and fruits. In this study, we found that the total per capita food consumption in three rural areas significantly increased. The per capita intake of poultry, sugar, egg, and meat greatly increased. However, meat has played an important role in people's daily diet, and it is not easy to change eating habits [[30], [31], [32], [33]]. It is worth noting that the study found an association between meat consumption and nine non cancer diseases, linking high meat consumption with negative health outcomes [34].The fifth Dietary Guidelines for Chinese Residents (2022) made a greater focus on the intake of whole grain foods, mainly due to the excessive amount of processed grains in the diet, emphasized eating aquatic products at least twice a week, with a particular emphasis on consuming one egg per day, proposed increasing the intake of milk and dairy products by 300–500 ml [35]. Healthy diet can help us to prevent the occurrence of malnutrition and non-communicable disease [36]. In recent years, people's intake of fat and carbohydrates has led to a series of diseases, which has large influences on public health [37,38]. The occurrence of obesity, diabetes, hyperlipidemia, hypertension and other chronic diseases is closely related to the dietary pattern, especially excess food energy intake [39]. These diseases not only have a long period of occurrence, but also easily lead to a series of complications [40]. The energy intake in the three rural areas increased gradually, especially in the Xinyang rural area. In the past decades, the excess intake of unhealthy foods has become a major public health problem [[41], [42], [43]]. From the perspective of energy balance in nutrition, continuous excessive energy intake inevitably leads to the accumulation of fat, which in turn leads to the occurrence of obesity. Some intervention measures must be planned in advance. By referring to areas with similar dietary habits in some developed regions or countries, we can predict problems in advance and learn some good experience to avoid the increase of incidence rate of related dietary metabolic diseases. It is widely agreed that people should reduce eating meat and increase their intake of vegetables and fruits [[44], [45], [46], [47], [48]]. Aquatic products have lower fat content compared to animal meat, and the fatty acids they contain are more conducive to protecting the cardiovascular system.

Many natural factors, such as geographical environment, ecological landscape and climate characteristics, can influence the food consumption of local people [49]. The rural residents prefer to be self-sufficient in some food, especially in grains, vegetables and poultry. To some extent, the high rate of food consumption is influenced by the endowment of local food resources. The food consumption of local residents will inevitably have a significant impact by the amount of production, what to plant more, what to eat more, and what to raise more. In Xinyang, rice is the main crop, and also the main diet. In Luoyang and Zhengzhou, rural residents prefer to wheaten food. Some social factors, such as eating habits and dietary culture, can influence the food consumption of local people. For example, the people in Xinyang are fond of stewed meat, so the consumption of meat would be much more than that of Luoyang and Zhengzhou. Income is an important economic factor that affects the amount and structure of food consumption. Food consumption is one of the key elements of environmental impact, accounting for approximately 20%–30 % when compared to the overall consumption statistics [50]. Food consumption may be influenced by many factors such as lifestyle, attitudes, and behaviors, as well as public health policies. However, how people connect food and food consumption is changing. The rise in obesity and related health concerns has shifted people's focus from the social aspects of food to its nutritional functionality [[51], [52], [53]]. In 2012, the per capita GDP of China was 4683.2 U S. dollar. In 2021, the per capita GDP of China was 8833.10 U S. dollar. In 2012, the per capita living expenditure of rural households was 748.20 U S. dollar. In 2021, the per capita living expenditure of rural households was 2092.40 U S. dollar. As the income of rural residents increases, the demand for various food types and consumption will also increase. After the energy sector, agricultural production is the industry that has the greatest impact on the environment [[54], [55], [56]]. Understanding the needs and trends of rural residents' food consumption can provide a detailed basis for the formulation of agricultural product supply policies.

Unsustainable diets are one of the main reasons for the nutrition–health–environment trilemma [[57], [58], [59]]. According to the prediction of The World Health Organization (WHO), the global burden of NCDs will rise from 49 % to 56 % by 2030 [[60], [61], [62]]. The important influencing factors are the shift in food consumption towards an increased intake of foods high in sugars, sweets, fats, and oils and a decrease in calorie expenditure [63,64]. Intensive meat production has brought negative external impacts on public health, environment, and animal welfare [65]. Taking pig farming as an example, intensive and large-scale farming has led to a high concentration of pig herds. The flushing water mixed with a large amount of urine and feces in the pigsty, the collected feces, the foul odor and volatile organic compounds released into the air by the environment will be polluted from multiple aspects such as air, water quality, and soil. Improper sewage treatment can also cause groundwater pollution, and untreated manure may also carry pathogenic microorganisms [66,67]. The relationship between intensive animal husbandry and ecological environment is not only a problem for the development of animal husbandry, but also an aspect of sustainable development of human society.

## 5 Conclusion

Through an analysis of food consumption in the three rural areas of Zhengzhou, Luoyang, and Xinyang, this study found that the food consumption of rural residents in Henan Province presented obvious characteristics.

(1) The total food consumption showed a significant increase, except for in 2020. The per capita consumption of poultry, meat, sugar, and egg (all of which are high-energy sources) by rural residents increased much more than that of other items, which means that the daily energy intake of rural residents has greatly increased.
(2) In the three rural areas, the proportion of grains, vegetables, liquor, and edible oils decreased overall. The proportion of other categories, such as poultry, meat, and fruits, increased. Through the study results, we found that rural residents' food intake is more diverse.
(3) The rural residents' per capita energy intake increased remarkably. The results suggest that rural residents should control their intake of high-energy food, such as poultry, meat, and sugar.

This research has achieved some meaningful points which could master the food consumption patterns and provide valuable strategy for local government members. At present, although rural areas have gained some knowledge in nutrition, there is still a lack of systematic advice. At the national level, major strategies have been proposed, but specific implementation still needs to rely on local governments. The government should take measures to lead rural households adopt sustainable food consumption practices and advance knowledge and awareness of sustainable food consumption pattern. Continuous data acquisition and analysis can help the government adjust policies dynamically, guide rural households to carry out sustainable food consumption behavior, and improve awareness of sustainable food consumption.

Based on the results of this study, it is suggested that Henan Province should increase the publicity and frequency of nutrition knowledge, so that farmers can receive correct and reliable nutrition advice. The concept of increasing the intake of aquatic meat and whole grain foods needs to be strengthened. However, there are still some shortcomings in this study due to the limitations of human and economic resources. Firstly, the lack of consideration for food waste is a limitation of this study. Secondly, this study only focused on the per capita energy and nutrient intake of rural residents, without paying attention to the population segmentation of rural residents, such as children and the elderly. Finally, we should also pay attention to the correlation between dietary changes and the incidence rate of some diseases. As more detailed interviewing information is necessary for this item study, and this will be our next important research target. In future, we will put more consideration into obtain more empirical data to further enrich our research content.

## Funding

This research was funded by the 10.13039/501100009101Key Research Projects of Higher Education Institutions in Henan Province (24B630009).

## Data availability statement

Data is contained within the article.

## CRediT authorship contribution statement

**Ping Wen:** Writing – review & editing, Writing – original draft, Validation, Resources, Project administration, Methodology, Investigation, Funding acquisition, Formal analysis, Conceptualization. **Na Zhu:** Visualization, Software, Formal analysis, Data curation. **Mengmeng Jia:** Validation, Supervision, Software, Conceptualization.

## Declaration of competing interest

The authors declare that they have no known competing financial interests or personal relationships that could have appeared to influence the work reported in this paper.
